# Semantically-Enhanced Feature Extraction with CLIP and Transformer Networks for Driver Fatigue Detection

**DOI:** 10.3390/s24247948

**Published:** 2024-12-12

**Authors:** Zhen Gao, Xiaowen Chen, Jingning Xu, Rongjie Yu, Heng Zhang, Jinqiu Yang

**Affiliations:** 1 School of Computer Science and Technology, Tongji University, Shanghai 201804, China; 2 Department of Computer Science, City University of Hong Kong, Hong Kong 999077, China; 3 Key Laboratory of Road and Traffic Engineering of the Ministry of Education, Shanghai 201804, China; 4 College of Transportation Engineering, Tongji University, Shanghai 201804, China; 5 Zhejiang Fengxing Huiyun Technology Co., Ltd., Hangzhou 311107, China; 6 Department of Computer Science and Software Engineering, Concordia University, Montreal, QC H3G 1M8, Canada

**Keywords:** fatigue detection, CLIP pre-trained model, Transformer, instance normalization, semantic analysis

## Abstract

Drowsy driving is a leading cause of commercial vehicle traffic crashes. The trend is to train fatigue detection models using deep neural networks on driver video data, but challenges remain in coarse and incomplete high-level feature extraction and network architecture optimization. This paper pioneers the use of the CLIP (Contrastive Language-Image Pre-training) model for fatigue detection. And by harnessing the power of a Transformer architecture, sophisticated and long-term temporal features are adeptly extracted from video sequences, paving the way for more nuanced and accurate fatigue analysis. The proposed CT-Net (CLIP-Transformer Network) achieves an AUC (Area Under the Curve) of 0.892, a 36% accuracy improvement over the prevalent CNN-LSTM (Convolutional Neural Network-Long Short-Term Memory) end-to-end model, reaching state-of-the-art performance. Experiments show that the CLIP pre-trained model more accurately extracts facial and behavioral features from driver video frames, improving the model’s AUC by 7% over the ImageNet-based pre-trained model. Moreover, compared with LSTM, the Transformer more flexibly captures long-term dependencies among temporal features, further enhancing the model’s AUC by 4%.

## 1. Introduction

Every year, drowsy driving, as reported by the National Safety Council (NSC) in the United States, leads to approximately 100,000 accidents, resulting in 1550 fatalities and 71,000 injuries, and contributes to about 9.5% of all accidents [1]. Fatigue is a major contributing factor that has heightened the occurrence of traffic accidents by diminishing drivers’ operational performance, mental alertness, and reaction time [2]. The implementation of an alert system for drowsy drivers could potentially prevent numerous accidents. Consequently, driver fatigue detection systems have garnered significant attention from researchers and scholars in recent years [3].

According to Chinese media reports [4], fatigue driving among long-distance passenger and freight vehicle drivers is widespread. A survey by relevant Chinese authorities showed that 84% of freight drivers exceed 8 h of driving per day, with 40% exceeding 12 h, and 64% of freight vehicles being operated by only one driver. To enhance driving safety, the Chinese government has mandated dynamic monitoring of operational vehicles and established fatigue driving thresholds: commercial drivers must not exceed 8 h of cumulative driving within 24 h, with a maximum of 4 h of continuous driving during the day and 2 h at night, and rest periods of no less than 20 min after driving [5]. Nonetheless, many drivers continue operating vehicles while fatigued due to workload pressures and a lack of awareness [3]. Consequently, it is necessary to establish a public management platform for long-distance passenger and freight vehicles, which can intelligently detect fatigue and automatically prompt drivers to correct such illegal behaviors [5].

Current approaches to drowsy driving detection primarily comprise four methods [1]: (1) individual-based, considering the driver’s sleep quality, mental state, and circadian rhythm [6]; (2) vehicle-based, focusing on lane deviation, steering wheel movement, speed, and braking patterns, etc. [7]; (3) video-based, using video sequences of drivers [8]; and (4) biological signal-based, involving sensors to monitor ECG (electrocardiogram), EEG (electroencephalogram) [9,10], and EOG (electrooculogram) [11]. Methods 1 and 2 are less accurate, while method 3, based on video-based driver behavior detection, offers higher precision but generally falls short of the accuracy of method 4. Nonetheless, due to its driver-friendly nature, lower cost, and ease of implementation, method 3 has become a focal point of recent research.

For fatigue detection methods based on video-based driver behavior detection, current research typically utilizes deep learning techniques to construct fatigue detection models. CNN networks [12] and LSTM networks [13] are extensively employed, with CNNs adept at extracting static features from video frames and LSTMs at capturing the temporal dependencies of these features. For these deep learning models, supervised learning is typically conducted based on driver video frames labeled with “fatigue” or “non-fatigue”, and model parameters are usually initialized with random values and then trained from the beginning. Ultimately, the accuracy of fatigue detection models relies not only on the learning capability of the deep neural networks themselves but also significantly on the quantity and quality of the annotated samples. Insufficient or poor diversity in the collected driver video data can adversely affect model quality.

Although deep learning models have made progress in fatigue detection, the difficulty in collecting facial video data from drivers, due to privacy concerns, has reduced model accuracy to some extent. Research notes that a common shortcoming is the insufficient number of driver participants, making it challenging to find a generalized model [14]. Current studies utilize public datasets with limited samples, such as YawDD [15] with 351 videos, NTHU-DDD [16] with 270 videos, and UTA-RLDD [17] with 180 videos. These public datasets were not collected during naturalistic driving but through elaborate designs, where drivers were instructed to perform certain actions (such as blinking, speaking, etc.) during video recording. Consequently, the authenticity of these data is inferior to that of naturalistic driving data. Some studies gathered naturalistic driving data, but the scale was not large enough, with ref. [18] collecting 2034 videos, ref. [19] using 2721 videos, and ref. [20] using 100 videos. Due to insufficient training samples, existing fatigue detection research often employs relatively lightweight network architectures like CNNs and LSTMs. More complex and capable architectures such as the Transformer [21], though widely adopted in various fields, have not been applied to video-based fatigue detection tasks to avoid overfitting on small datasets.

Recently, some pre-trained models based on self-supervised learning, such as BERT and Chat-GPT, have been developed rapidly, addressing the data scarcity and difficult labeling issues in supervised learning. As a new pre-trained model, CLIP (Contrastive Language-Image Pre-Training) [22] has shown impressive zero-shot transfer capability across various domains. CLIP (Contrastive Language-Image Pre-training) is a large-scale model that has been trained on an extensive dataset of 400 million image–text pairs collected from the web. It extracts image features under the guidance of textual semantics, enabling the model to learn more general visual semantics. CLIP significantly outperforms widely used visual encoders trained with in-domain annotated data [23]. Therefore, utilizing CLIP as the backbone network for fatigue detection can enhance the extraction of more generalized behavioral features. Additionally, a vast dataset is gathered, comprising over 25,000 driver videos recorded during naturalistic driving conditions, with a significant portion containing over 2000 videos of drowsy driving. Based on the large dataset, the complex Transformer network could be used to extract long-term dependencies between the visual features of video frames, with the aim of identifying prolonged driver behavioral patterns such as frequent yawning for accurate fatigue detection.

In summary, this paper proposes a novel fatigue detection method that employs CLIP as the backbone network to extract generic features from driver facial video frames (such as eye closure and mouth opening), followed by using a Transformer network to capture long-duration fatigue behavioral characteristics (such as prolonged eye closure and frequent yawning). The combination of technologies, which uses CLIP for feature extraction and transformers for classification tasks, has significant advantages [24]. To the best of our knowledge, this is the first time that both the CLIP pre-trained model and Transformer are applied to the fatigue detection task based on driver’s facial video data.

To summarize, the key contributions are outlined as follows:

(1) This study is the first to apply the pre-trained model CLIP to fatigue detection, utilizing it to extract generic features of driver faces and behaviors. Compared with models trained from scratch, CLIP can leverage its zero-shot transfer ability to identify a richer set of driver behaviors and enhance model interpretability by using textual semantics.
(2) The Transformer is innovatively utilized to capture the temporal patterns of drowsy driving. With the assistance of instance normalization, which filters out invariant features across video frames, it enhances the accuracy of fatigue detection.
(3) This study presents a novel, end-to-end modeling approach called the CLIP-Transformer Network (CT-Net), which utilizes in-vehicle facial video recordings to detect driver fatigue in real time. The proposed CT-Net has demonstrated state-of-the-art (SOTA) performance.

The organization of the paper is as follows: Section 2 presents a literature review. Section 3 describes the data sources. Section 4 elaborates on the methodology and model architecture. Section 5 details the experimental results and ablation experiments. Section 6 discusses the advantages of the pre-trained models and explores the feasibility of applying CT-Net in high-risk escort scenarios. The final chapter offers a conclusion and mentions future works.

## 2. Related Works

The data sources for fatigue detection mainly include individual driver information (such as driver sleep and mental state and circadian rhythm [6]), vehicle kinematics and maneuver data [7] (such as speed, acceleration, braking patterns, pressure on the accelerator, steering wheel patterns, deviations from lane position, etc.), driver physiological data (such as electroencephalogram signals [25]), eye-tracking data [26], and drivers’ behavior (such as continuous blinking, head movements, yawning, etc.) [8].

However, most sources present their own challenges. Individual driver information, such as sleep patterns, mental state, and circadian rhythms [6], which are inherently subjective and exhibit individual variability, limiting their effectiveness in consistent fatigue detection. For vehicle kinematics and maneuver data, like speed, acceleration, braking patterns, and steering wheel patterns, as well as deviations from lane position [7], are indicative of potential fatigue, with lane departure [27] and steering wheel angle [28] being particularly telling signs. However, these indicators can be unreliable due to variations in driving styles and the influence of road geometry, such as on steep and uneven roads [1]. Regarding driver physiological data, including electroencephalogram signals [25], offer a higher level of reliability and efficiency but are often impractical due to discomfort from invasive sensors and sensitivity to noise [29]. Furthermore, eye-tracking data, while valuable, faces challenges in implementation due to the high cost of advanced systems and the technical requirements for high-speed cameras and processing capabilities [30].

In contrast, driver behavior-based approaches using in-vehicle video have emerged and been widely used as effective, non-invasive, and cost-effective methods for fatigue detection. With the advancement of AI technology, the automatic analysis of driver video data through various deep neural network (DNN) methods has become the mainstream approach, achieving high accuracy and becoming increasingly prevalent in the field of fatigue detection [13,31].

Combining explicit features with rules for fatigue assessment can achieve high detection accuracy. For instance, rule-based methods typically calculate parameters such as PERCLOS (PERcentage of eyelid CLOSure over the pupil over time) [32] and yawn frequency based on facial keypoints detected by some computer vision algorithms. However, these rule-based methods heavily rely on the accuracy of facial keypoint detection algorithms. But these algorithms have inherent flaws. For instance, the DLIB algorithm [33] can fail to detect when the driver turns his head at a large angle, and the SDDFAv2 [34] algorithm is insensitive to closed-eye detection. Furthermore, in complex real-world driving environments, factors such as strong lighting, facial occlusion, and wearing glasses will reduce the accuracy of keypoint detection, ultimately leading to algorithm failure [19]. These rule-based methods have low fault tolerance, and errors in keypoint detection can directly lead to algorithm failure, resulting in poor robustness in real-world environments.

Early deep learning methods, predominantly based on the combination of explicit features and fatigue assessment rules, have demonstrated efficacy in driver fatigue detection [3]. Most of them typically extract various behavioral indicators, including eye-related features such as blinking frequency [35], PERCLOS [32], eye-open degree [36], and eye position [37], as well as mouth-related features like yawning [38], head position [37], facial expressions [39], multi-facial features [40], and even hand movement features, etc. These studies collectively affirm that integrating explicit features with fatigue assessment rules can indeed achieve high detection accuracy, showcasing the potential of this approach in driver fatigue detection systems.

As a result, end-to-end approaches have emerged, utilizing deep neural networks to autonomously extract relevant features for evaluating fatigue levels. Model visualization interpretations indicate that this approach can effectively consider eye, mouth, head, and driver limb movements for fatigue assessment, demonstrating greater robustness in diverse real-world driving environments [19].

For end-to-end models, accurate extraction of high-level image features is crucial for precise fatigue identification. Current research primarily focuses on extracting these features using CNN networks. CNN, or multitasking CNN, ref. [41] is commonly used for extracting static image features from single video frames, such as eye closure and mouth opening features. Spatiotemporal feature learning is better suited to three-dimensional processes, so frame-based and multistep 3D CNNs [42] are widely used for drowsiness detection. Various DCNN architectures, including VGG-FaceNet, AlexNet, FlowImageNet, and ResNet, have been tried [39], and additional DCNN models, such as Xception, InceptionV4, and ResNext101, have also been verified to be effective for fatigue detection [20]. However, current end-to-end fatigue recognition models may suffer from incomplete or coarse-grained extraction of high-level features. For example, these models may only focus on the contour features of the eyes and mouth, overlooking detailed representations of mouth movements, thus failing to distinguish actions such as eating, yawning, or speaking. Additionally, the bodily actions typically employed by drivers to counter weariness, like rubbing their eyes or shaking their heads, are likely to be infrequently identified due to the lack of collection of relevant samples. So, new methods are needed to extract more detailed and comprehensive driver behavior features, and the development of new technologies is still necessary to further enhance the accuracy and reliability of these systems in the future [3].

The introduction of CLIP (Contrastive Language-Image Pre-Training) in 2021 has provided feasibility for extracting more fine-grained and comprehensive general behavioral features from images. CLIP, trained on 400 million image–text pairs, has quickly achieved state-of-the-art results in various fields. Unlike task-specific networks, such as image classification or fatigue recognition, CLIP does not have a specific task. Instead, it utilizes contrastive learning with paired image–text samples under the guidance of textual semantics to extract visual features, enabling it to learn more general visual semantics and possess strong zero-shot transfer ability. The CLIP consists of two main components: an image encoder and a text encoder. The pre-trained image encoder can learn fine-grained visual concepts and transfer knowledge for retrieval tasks [43]. Additionally, CLIP exhibits improved robustness, as indicated by Radford et al. [22], who noted that CLIP closes the “robustness gap” by up to 75% while matching the performance of the original ResNet-50 on ImageNet zero-shot without using any of the original 1.28 million labeled examples. In fact, robustness represents a significant weakness of deep learning algorithms and must be taken seriously. Pham [44], Yang [45] and Peng [46] have explicitly pointed out this in their research on autonomous driving systems. Therefore, it is worth considering the use of CLIP pre-training models in fatigue detection models to extract more fine-grained and extensive behavioral features from drivers.

Fatigue, as a prolonged behavioral pattern, requires analysis over a sequence of video frames to accurately detect. While a single instance of a driver’s eye closure may be normal, a consistent closed-eye state across multiple frames can indicate a state of shallow sleep, posing a significant risk to driving safety. To address this, researchers have been developing methods that leverage advanced computational models for drowsiness detection. For example, Chen proposed methods using factorized bilinear feature fusion and long short-term recurrent convolutional networks [47]. In another study, Quddus et al. suggested a driver drowsiness detection approach using a combination of convolutional neural networks (CNN) and long short-term memory (LSTM) networks in a simulated driving experiment [13]. Gao further enhanced this by proposing a two-stage feature extraction method that combines CNN with LSTM networks to accurately evaluate the fatigue state from consecutive frames [19].

To capture not only momentary but also long-term dependencies in driver behavior, such as “frequent blinking”, it is essential to employ models that excel in understanding temporal dynamics. The Transformer model, introduced by Vaswani in 2017 [21], has demonstrated exceptional ability in capturing long-term dependencies, often outperforming RNN/LSTM models. Through its self-attention mechanisms, the Transformer effectively utilizes the correlation within input sequences, offering faster computational speeds. Its versatility has led to widespread adoption across various domains, including text, speech, and image processing. Despite the Transformer’s success, there has been a gap in its application for detecting driver fatigue from video data. This study aims to bridge that gap by harnessing the Transformer’s multi-head attention mechanism to extract multi-dimensional, long-term behavioral features indicative of drowsy driving.

In conclusion, for fatigue detection based on driver facial videos, it is essential to explore advanced end-to-end deep learning modeling methods to further improve the accuracy of the model. This study proposes the use of the CLIP language-image pre-trained model to extract comprehensive, fine-grained features of momentary driving behaviors, including eye closure, yawning, phone usage, frowning, smiling, raising a hand, eating, etc. These features will then be sequentially input into a Transformer network, leveraging its powerful self-attention mechanism to establish temporal correlations among these momentary features and extract long-term behavioral features such as prolonged eye closure and frequent yawning as the basis for fatigue assessment, aiming to enhance the model’s accuracy. In addition, CLIP’s ability to convert text information into a linguistic view of images greatly improves the interpretability of the model. To the best of our knowledge, this is the first application of the CLIP pre-trained model and Transformer in the task of fatigue detection based on driver video data.

## 3. Data Source

Multiple fatigue detection studies were conducted in a virtual or simulated environment, and consequently, the findings may not always accurately reflect real-world driving conditions. Therefore, real-world autonomous driving data were used for this study.

The paper utilizes data from a large logistics company, where freight drivers are occupied with either short-haul or long-haul transportation duties. All vehicles are equipped with in-vehicle cameras, which capture facial videos of drivers during naturalistic driving. These videos are analyzed in real time using computer vision algorithms to detect driver states, such as frequent blinking and yawning. Upon detecting frequent eye closure, yawning, or other similar behaviors, the Driver Monitor System (DMS) will trigger an alarm and capture the current 6-s video segment to upload to the cloud. Due to the limitations of the on-board DMS algorithm, there is a high false alarm rate, and it is not yet able to intelligently identify fatigue and non-fatigue states. These 6-s driver videos were collected from the cloud to build a fatigue detection model.

The training dataset consists of 25,000 driver video records, with a resolution of 1280 × 720 and a frame rate of 15–25 frames per second. It comprises 4851 vehicles, gathered between 22 April 2022 and 22 July 2022. The driver video collection environment is diverse, including various weather conditions (sunny, rainy, cloudy) and different lighting conditions (daytime, nighttime), encompassing videos in strong sunlight as well as low-light nighttime conditions. The camera angles vary, with most capturing the driver’s face in the center of the frame, while some only capture one side of the driver’s face. Additionally, some drivers wear masks covering their mouths, some wear sunglasses covering their eyes, and some wear nearsighted glasses experiencing reflective phenomena in strong light, all of which add complexity to fatigue detection.

Random sampling was conducted on the training dataset, using 80% for training, 10% for validation, and 10% for testing.

Based on expert experience and incorporating the Karolinska Sleepiness Scale (KSS) questionnaires in Table 1, which is a widely used nine-level scale for assessing drowsy driving [48], the driver videos were classified into two different schemes. In the first scheme, videos are categorized into fatigue and non-fatigue, with KSS levels one–six classified as non-fatigue and seven–nine as fatigue. The second scheme includes three labels: non-fatigue, fatigue, and high-risk, with KSS levels one–five classified as non-fatigue, six–seven as fatigue, and eight–nine as high-risk.

## 4. Methodology

### 4.1. Model Architecture

**Figure 1:**
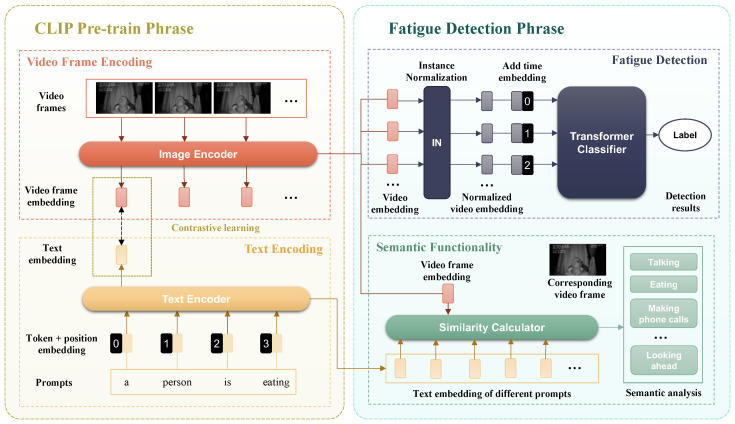
CT-Net architecture.

The CT-Net (CLIP-Transformer Network) model serves two main purposes: (1) fatigue detection and (2) semantic explanation. Structurally, the network can be divided into two components: the CLIP pre-trained basic visual feature extraction network and the Transformer network for extracting temporal features to detect fatigue states. The architecture of the CT-Net network is illustrated in Figure 1.

### 4.2. CLIP Pre-Train Phrase

CLIP (Contrastive Language-Image Pre-training) is a contrastive learning-based multi-modal model that utilizes image–text pairs for pre-training. CLIP comprises two components: the Image Encoder, which extracts features from images using network architectures such as ResNet or ViT (Vision Transformer), and the text encoder, which extracts features from text using commonly used NLP architectures like CBOW or text transformer.

The loss function for the image encoder is denoted as *loss_i*, and for the text encoder as *loss_t*. The overall CLIP loss function, denoted as loss, is computed using *f* for cross-entropy calculation and *s* for cosine similarity calculation.

(1) loss_i=f(s(IE(image),TE(text)),labels)

(2) loss_t=f(s(TE(text),IE(image)),labels)

(3) $$loss=\frac{1}{2}\left(loss\_i+loss\_t\right)$$

where *IE* represents the output features from the image encoder and *TE* represents the output features from the text encoder. The two tasks of CT-Net, detecting driver fatigue and interpreting driver behavior semantically, are carried out by the fatigue detector and semantic interpreter, respectively.

### 4.3. Fatigue Detector

For the design of the fatigue detector, in order to enhance the model’s ability to represent general driver behavior features, the image encoder of CLIP, utilizing the ResNet50 model architecture, is directly adopted to extract the visual features (1024-dimensional feature vectors) of each frame of driver videos.

To intensify the model’s focus on the correlation of visual features within the same video frames, CLIP modifies the original ResNet50 model’s final output layer by substituting the AvgPool with an AttentionPool, which leverages a multi-head self-attention mechanism and position embedding to precisely detect the driver’s multi-dimensional, fatigue-indicative sequential features, such as frequent blinking and yawning.

The AttentionPool first encodes the vectors output from the earlier layers of ResNet50 using position embedding and then feeds them into the multi-head attention. In each head, the input vectors are first multiplied with a weight matrix to obtain *Q* (Query), *K* (Key), and *V* (Value). The components *Q*, *K*, and *V* are instrumental in the multi-head self-attention mechanism, which is a cornerstone of the model’s ability to learn cross-modal representations. They are derived from the input data through linear transformations, where the weights are learned during training. *Q* represents the input features that the model is currently focusing on to gather relevant information. *K* acts as a filter to determine which parts of the input data are most relevant to the Query. And *V* contains the actual data or features that will be weighted and combined based on the attention scores. Subsequently, the multiplication of *K* and *Q*, passed through a mask and a Softmax layer, is then multiplied with *V* to obtain the output features.

(4) $$Attention\left(Q,K,V\right)=softmax\left(\frac{QK^{T}}{\sqrt{d_{k}}}\right)V$$

Finally, concatenating the features outputted by multiple heads yields a 1024-dimensional feature for a single-frame image.

(5) $$MultiHead\left(Q,K,V\right)=Concat\left(head_{1},\cdots ,head_{h}\right)W^{O}$$

where

(6) $$head_{i}=Attention\left(QW_{i}^{Q},KW_{i}^{K},VW_{i}^{V}\right)$$

If the input video segment is represented as V(T×C×H×W), which is a sequence of image frames in the video segment, where *T* represents the number of video frames, *C* represents the number of image channels, *H* represents the image height, and *W* represents the image width, passing multiple frames of a video through the Image Encoder sequentially yields T×1024 features.

The T×1024 features obtained from the ResNet50’s output are then input into a Transformer to extract long-term temporal behavioral features of the driver and subsequently fed into an MLP classifier to output the probability of fatigue/non-fatigue. To capture the changing features of the video frames (such as the opening and closing of eyes/mouth) while ignoring constant features (such as vehicle interior background and driver attire), instance normalization is applied to normalize the data between multi-frame features.

The detailed processing workflow for the fatigue detector is as follows:

- Visual feature extraction: the video frames are used as input to the image encoder, which outputs the visual representation of the driver’s video frames.
- Normalization processing: the visual representation undergoes instance normalization to filter out invariant features across video frames, including driver appearance, attire, seat decoration, etc., and a positional embedding is added to each frame to provide temporal information.
- Sequential feature extraction: a Transformer-based classifier is used to encode the processed visual features of each frame and analyze their temporal dependencies, resulting in the high-level video representation.
- Classification: a fully connected layer is employed to obtain the fatigue probability as output, with the high-level video representation serving as input.

The fatigue detector is trained based on the training dataset, with the CLIP image encoder ResNet50 network parameters being fine-tuned, while the Transformer and MLP parameters are trained from scratch.

### 4.4. Semantic Interpreter

The semantic interpretation of driver behavior is primarily achieved through the CLIP text encoder. During CT-Net training, the text encoder is kept unchanged. During model inference, the video frame data are sequentially input into the fatigue detector to output fatigue/non-fatigue probabilities. Simultaneously, various descriptions of driver behaviors, such as closing eyes and yawning, etc., are deliberately crafted as prompts and fed into the CLIP text encoder to extract high-level textual representations, which are then used to compute cosine similarities with the high-level visual features generated by the fatigue detector’s image encoder. This process enables the determination of the likelihood of specific driver behaviors, such as looking ahead or yawning, ultimately contributing to the semantic understanding of the video.

The specific steps include the following:

- Visual feature extraction: using the video frames as input to the image encoder, the visual features of the driver’s video frames are outputted.
- Textual feature extraction: for the text encoder, the deliberately designed prompts are inputted to obtain textual representation.
- Similarity calculation and semantic analysis: A semantic output layer is employed to determine the confidence level of possible driver behaviors as described by the prompts. The input consists of visual features *a* and textual features *b*, with the output being the cosine similarity coefficient calculated in the following manner:
  (7) $$cos\left(\theta \right)=\frac{a\cdot b}{\left||a||||b|\right|}$$

In terms of the model’s semantic output, the input text, known as a prompt, plays a crucial role. The prompts can be manually designed, such as “A person class”. Based on prior knowledge, the prompts were manually designed for the video frame, as shown in Table 2, which categorizes the driver’s state into nine classes, including eye state, mouth state, body movements, facial expressions, and other content. Each token for the prompt has multiple possible candidates, with the maximum probability value being assigned as the token’s value (e.g., closed eyes). The comprehensive semantic output of the video comprises the corresponding prompts outlining driver behavior, along with the duration of those actions (for instance, a driver closed their eyes for a duration of 2 s).

The token, combined with the prompt to form a sentence, undergoes processing through the position embedding layer and token embedding layer of the text encoder, resulting in contextual features. These features are then input into a 12-layer Transformer, where each residual layer consists of a multi-head self-attention (MHSA) mechanism and a feed-forward network (FFN). The input vectors, after passing through these layers, are then merged with the original inputs. The formula is as follows:

(8) ResidualAttention(x)=Concat(y,MHSA(y))

where

(9) y=Concat(x,FFN(x))

## 5. Experiments and Results

### 5.1. Model Accuracy Evaluation

A total of 25,000 6 s videos were collected and randomly allocated: 80% for training, 10% for validation, and 10% for testing. CT-Net training was stopped based on the performance on the validation dataset. Performance evaluation was conducted on the test dataset, with the CNN-LSTM (Convolutional Neural Network-Long Short-Term Memory) model from the paper [12,13,19] serving as the baseline model.

Additionally, several common metrics are utilized from classification tasks to evaluate the performance of the proposed model. These include model accuracy, which measures the proportion of correct predictions made by the model; recall, indicating the ability of the model to find all relevant instances within the dataset; precision, which assesses the accuracy of the positive predictions made by the model; and the F1 score, a harmonic mean of precision and recall that provides a balance between the two. Lastly, the term “semantic” in this context refers to the model’s capacity for semantic interpretability, reflecting its ability to provide meaningful insights into the data it processes. The experimental results are shown in Table 3.

**Figure 2:**
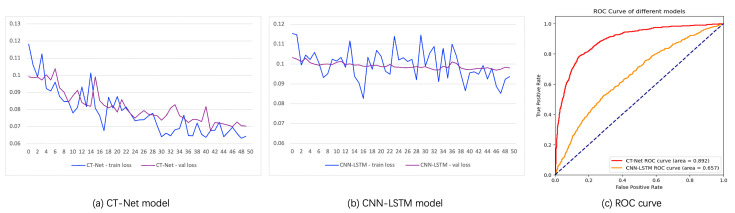
Loss function curve of the CT-Net model and the baseline model.

The loss function curves of CT-Net on the training and validation datasets, as shown in Figure 2a, indicate low training and validation losses, demonstrating successful convergence of the network. In contrast, the loss function curves of the CNN-LSTM model on the training and validation datasets, depicted in Figure 2b, indicate convergence, but the high training and validation losses suggest model underfitting. The ROC curves and performance metrics of the CT-Net and CNN-LSTM models on the test dataset are presented in Figure 2c and Table 3, revealing that the CLIP-Transformer model not only possesses semantic capabilities absent in the CNN-LSTM model but also exhibits significantly superior accuracy.

In the paper [19], the CNN-LSTM model was trained on 1632 samples, achieving an AUC (Area Under the Curve) of 0.92. In the experiments, the model was trained on 20,000 samples, resulting in a CNN-LSTM model AUC of 0.657. The substantial disparity between the results and those of the paper [19] is likely attributed to the difference in training dataset size. The smaller dataset used in the paper [19] justified the reasonable design choice of training a lightweight CNN from scratch. However, as the dataset size increased, fatigue features became more complex and diverse, surpassing the capability of lightweight CNN models to extract more complex features, leading to underfitting. This may constitute the primary reason for the poor performance of the CNN-LSTM model in the experiment. Thus, when designing network architectures, dataset size should be a crucial consideration.

### 5.2. Ablation Experiment

To validate the advantages of the instant visual feature extraction network (CLIP pre-trained image encoder) and the long-term behavior feature extraction network (Transformer) in the CT-Net model, an ablation experiment is conducted. The experiment comprehensively compared the performance metrics of the following models (as shown in Table 4).

Model 1 represents the proposed final model, CT-Net, with the learning rate for the CLIP pre-trained image encoder (ResNet50) set to 0.00001 and the learning rate for the Transformer set to 0.0001. Model 2 replaces the first part of CT-Net’s CLIP image encoder with the ResNet50 pre-trained model trained on ImageNet, with the learning rate set to 0.00001 and 0.0001 for the Transformer, respectively. Model 3 replaces the detector (Transformer) of CT-Net with an LSTM model. Model 4 combines the replacements from Model 2 and Model 3. The performance of these models on the same testing dataset is presented in Table 4.

From Table 4, it is evident that replacing the CLIP pre-trained model in CT-Net with the ImageNet pre-trained model results in a decrease in AUC by 0.056, indicating that the image features obtained from the CLIP pre-trained model exhibit better transferability than those from the ImageNet pre-trained model. Furthermore, keeping the CLIP pre-trained model unchanged in CT-Net and replacing the Transformer with LSTM leads to a decrease in AUC by 0.034, suggesting that the Transformer is more flexible in capturing long-term dependencies between features, thereby achieving higher accuracy. Finally, if both the CLIP pre-trained model and the Transformer are replaced simultaneously, the model’s AUC decreases by 0.081. The CT-Net design effectively improves the accuracy of fatigue detection.

### 5.3. Semantic Analysis

**Figure 3:**
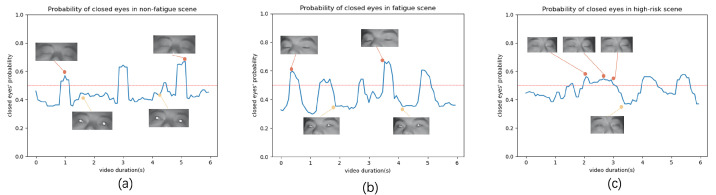
Semantic interpretation utilizing eye state-related prompt. (**a**) The change in probability of an eye-closing event that occurs during normal driving. (**b**) The change in probability of an eye-closing event that occurs during fatigued driving. (**c**) The change in probability of an eye-closing event that occurs during highly fatigued driving.

The CT-Net is featured for its dual capabilities: detecting driver fatigue and, impressively, providing a descriptive portrayal of driver behavior via prompts. Taking driver eyes state description as an example, with tokens “opened eyes” and “closed eyes”, the semantic interpretation is illustrated in Figure 3, where the *x*-axis represents video time and the *y*-axis represents the probability of “A driver closed eyes”. A probability y > 0.5 indicates that the model identifies the driver as being in a closed-eye state. Figure 3a depicts a sample of a driver in normal driving condition, showing regular, rapid blinking actions, indicating a non-fatigued state. Figure 3b depicts a mildly fatigued driver, with frequent and slow blinking actions lasting close to 1 s, indicating the onset of fatigue. Figure 3c shows a heavily fatigued driver with prolonged and frequent closed-eye intervals, some lasting close to 2 s, indicative of severe fatigue.

**Figure 4:**
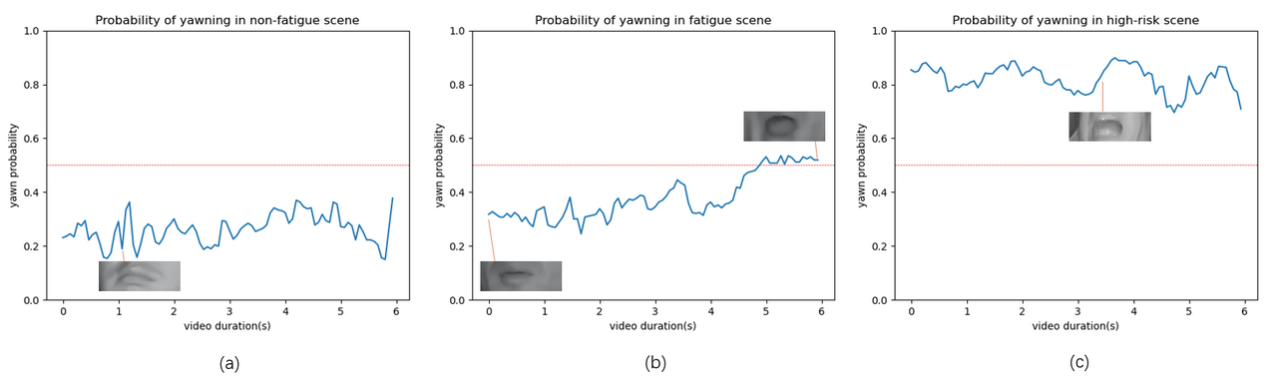
Semantic interpretation utilizing mouth state-related prompt. (**a**) The change in probability of a yawn event that occurs during normal driving. (**b**) The change in probability of a yawn event that occurs during fatigued driving. (**c**) The change in probability of a yawn event that occurs during highly fatigued driving.

Similarly, considering driver mouth state, with tokens “yawn” and “did not yawn”, the semantic interpretation is depicted in Figure 4. Figure 4a shows a sample of a driver in a normal driving condition, with a yawn probability curve indicating that the driver remains mostly in a closed-mouth state. Figure 4b illustrates a mildly fatigued driver, with a slight yawn observed towards the end of the video, suggesting the onset of mild fatigue. Figure 4c portrays a heavily fatigued driver, with continuous yawning, indicating heavy fatigue.

**Figure 5:**
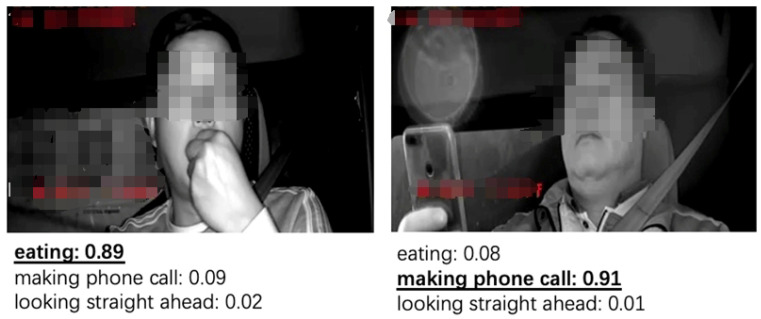
Semantic interpretation utilizing some behavior-related prompt.

In addition to retrieving semantic insights regarding the states of eyes and mouths, CT-Net can also accurately recognize behaviors such as eating and talking on the phone (as shown in Figure 5), which can further assist in determining the driver’s fatigue status; for instance, drivers typically do not experience fatigue while eating or talking on the phone. In this case, the prompt was designed as follows: A driver description, where the description could be “eating”, “making phone call”, or “looking straight ahead”.

These comprehensive experimental results clearly illustrate that CT-Net not only precisely evaluates fatigue levels but also effectively retrieves driver behavior information through prompts, thereby highlighting its proficiency in both fatigue prediction and semantic interpretation.

## 6. Discussion

This section primarily discusses and validates the advantages of the CLIP pre-trained model used in CT-Net.

### 6.1. Leverage of Pre-Trained Models

To illustrate the advantages of the pre-trained model, comparative experiments were conducted. Experiment 1 utilized the ResNet50 model, training the network parameters from scratch. Experiment 2 involved training the pre-trained model of CLIP ResNet50 with fixed parameters, during which only the parameters of other networks except for CLIP ResNet50 were updated. Experiment 3 involved training with the CLIP ResNet50 pre-trained model, updating the ResNet50 network parameters based on fatigue labels for fine-tuning. In a nutshell, Experiment 1 involved training the model from scratch without a pre-trained model. Experiment 2 utilized the fixed pre-trained model entirely, and Experiment 3 fine-tuned the pre-trained model based on fatigue labels.

**Figure 6:**
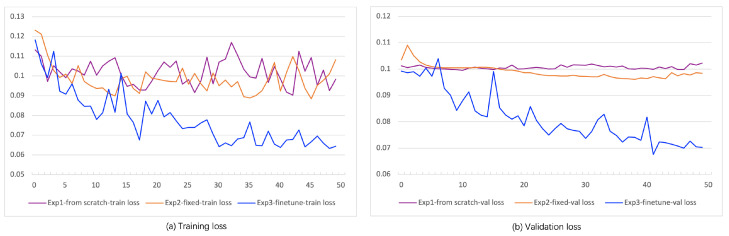
Training and validation loss curve of three experiments.

The loss function curves of these models during training are depicted in Figure 6a,b. Experiment 1 showed slow learning when trained from scratch, with significant oscillation in training loss (in Figure 6a) and consistently high validation loss (in Figure 6b), indicating underfitting. Experiment 2 exhibited lower and decreasing validation loss compared with Experiment 1, yet the final loss remained relatively high, likely due to the limited ability of the general visual features extracted by CLIP to reflect fatigue features, resulting in a higher training loss and suboptimal model accuracy. In contrast, Experiment 3 demonstrated the fastest convergence and achieved the lowest loss, maintaining it around 0.070, representing the best model accuracy. These experiments collectively indicate that employing a pre-trained model can aid in better extracting facial and behavioral features of drivers, and fine-tuning the CLIP pre-trained model based on a fatigue-labeled training dataset enables the model to concentrate more effectively on fatigue-related behavioral features rather than general behavioral features, thus achieving optimal accuracy.

### 6.2. Performance of CT-Net in High-Risk Escort

To balance the model’s predictive effects across different fatigue levels, appropriate classification thresholds need to be calculated. The labels are adjusted from “fatigue”/“non-fatigue” to “high-risk”/“fatigue”/“non-fatigue” and the Youden index method [49] was employed to calculate the optimal classification thresholds. The model’s confusion matrix on the testing dataset is presented in Table 5, with performance metrics shown in Table 6. The average AUC across different fatigue levels is 0.8740, with the model accuracy at 81%. Out of 218 instances of high-risk drowsy driving behaviors, 72% were accurately identified, indicating the model’s strong capability in detecting high-risk driving behaviors. Moreover, among 2065 non-fatigue driving instances, only 7% (1–93%) were misclassified, maintaining the false alarm rate for drowsy driving at a relatively low level. The experimental results demonstrate that the CT-Net model can be applied for high-risk escort applications.

## 7. Conclusions and Future Work

For the assessment of driver fatigue, the CT-Net model is proposed, which represents the first attempt to apply the CLIP image–text pre-training model (ResNet50) to fatigue detection. This model extracts instantaneous behavioral features of the driver and achieves state-of-the-art (SOTA) performance, with a 7% increase in the AUC for fatigue detection compared with the traditional ImageNet pre-trained ResNet50 model. Additionally, the application of the Transformer is introduced for fatigue assessment, which is used to extract temporal dynamic features of video frames. Compared with the commonly used LSTM modeling method in existing research, this approach results in a 4% increase in the AUC for fatigue detection. Furthermore, when compared with the CNN-LSTM end-to-end model in existing research, the CT-Net model shows a 10% increase in AUC. The introduction of the CLIP image–text pre-training model endows the CT-Net model with semantic functionality, thereby enhancing its interpretability and enabling real-time interpretation of driver states.

The CLIP-Transformer network architecture proposed is versatile, and the CLIP model introduced by CT-Net possesses universal visual representation capabilities, enabling it to learn diverse visual concepts. This not only improves its performance in fatigue detection tasks but also facilitates rapid support for new tasks, thereby enhancing the interpretability and credibility of DNN models.

This work, while presenting a novel approach to fatigue detection using the CLIP language-image pre-training model, is not without its limitations. One primary limitation is the reliance on hand-crafted prompts for semantic interpretation, which may not fully exploit the model’s semantic capabilities. Future studies could benefit from exploring the automatic generation of prompts, potentially enhancing the model’s ability to understand and interpret context. Additionally, the choice of the CLIP model for pre-training, while effective, may not be the optimal choice for video-based data. The adoption of a language-video pre-training model in future work could lead to faster training times and improved accuracy, aligning more closely with the nature of the data.

Furthermore, it is recommended that future research should address the scalability and generalization of the model across various datasets and real-world scenarios to ensure its robustness in different environments. Lastly, considering the computational constraints, there is a clear need for research into optimizing model training under limited resource conditions, possibly through more efficient architectures or innovative training strategies. By addressing these limitations and following these recommendations, it is believed that future work can significantly contribute to the advancement of fatigue detection systems and semantic analysis in video data.

## Figures and Tables

**Table 1:** KSS questionnaires.

Scale	Description
1	Extremely alert
2	Very alert
3	Alert
4	Fairly alert
5	Neither alert nor sleepy
6	Some signs of sleepiness
7	Sleepy, but no effort to keep alert
8	Sleepy, with some effort to keep alert
9	Very sleepy, with great effort to keep alert

**Table 2:** Prompt design.

Prompt	Token
A driver {class}	[“closed eyes”, “opened eyes”]
	[“yawn”, “did not yawn”]
	[“ate food”, “did not eat food”]
	[“drunk water”, “did not drink water”]
	[“made phone calls”, “did not make phone call”]
	[“sent message”, “did not send message”]
	[“talked”, “did not talk”]
	[“laughed”, “did not laugh”]

**Table 3:** Performance comparison between the CT-Net model and the baseline model.

Model	AUC	Accuracy	Recall	Precision	F1	Semantic
CNN-LSTM [12,13,19]	0.657	62.8%	58.2%	34.9%	0.436	No
CT-Net (our)	**0.892**	**84.0%**	**78.5%**	**64.6%**	**0.709**	**Yes**

**Table 4:** Performance of different models.

Model Name	Encoder	Detector	AUC
CT-Net (our)	CLIP	Transformer	0.892
CT-Net w/o CLIP	ImageNet	Transformer	0.836
CT-Net w/o Transformer	CLIP	LSTM	0.858
CT-Net w/o (CLIP and Transformer)	ImageNet	LSTM	0.811

**Table 5:** Confusion matrix.

Predicted Ture-Label	Non-Fatigue	Fatigue	High-Risk	Total
Non-fatigue	1912	74	79	2065
Fatigue	195	147	119	461
High-risk	50	12	156	218
Total	2157	233	354	2744

**Table 6:** Performance metrics of high-risk escort model.

Label	Precision	Recall	F1	AUC
Non-fatigue	89%	93%	0.91	0.8902
Fatigue	63%	32%	0.42	0.8227
High-risk	44%	72%	0.55	0.9089
Average	65%	65%	0.62	0.8740

## Data Availability

Because of privacy concerns, the research data in the form of driver videos cannot be provided in this paper.

## Abbreviations

The following abbreviations are used in this manuscript:

CLIP	Contrastive Language-Image Pre-training
CNN	Convolutional Neural Networks
LSTM	Long Short-Term Memory
ROC	Receiver Operating Characteristic
AUC	Area Under Curve
